# Lending a Hand-Over: Implementing a Digital Handover System Across North Glasgow Mental Health Hospitals

**DOI:** 10.1192/bjo.2026.11512

**Published:** 2026-06-30

**Authors:** Aized Raza Shahbaz, Fraser Walker, Alasdair Philbey, Nancy Liu, Sophie Flood

**Affiliations:** 1 NHS Greater Glasgow and Clyde, Glasgow, United Kingdom; 2 University of Glasgow, Glasgow, United Kingdom

## Abstract

**Aims:**

This quality improvement project sought to design and implement a new handover system for mental health hospitals in North Glasgow. The new system aimed to improve ease of access and editing, alongside reducing the frequency of missed tasks for resident doctors.

**Methods:**

Resident doctors across Gartnavel Royal Hospital and Stobhill Hospital were surveyed (cycle 1) between 06/06/25 and 13/06/2025 regarding their opinions on their respective existing Microsoft Excel handover systems, as well as the frequency of missedtasks and their willingness to try a new system. A new system was designed for each hospital using the Planner task management software on Microsoft Teams, adapted from a similar successful pilot in South Glasgow. Training sessions were delivered over June and July 2025, and the new systems were implemented on 18/07/2025. Resident doctors were surveyed again (cycle 2) between 04/08/2025 and 12/08/2025 regarding their opinions on the new system, any missed tasks and whether they wished to keep the new system or revert to the previous one. Adjustments to systems were made as per respondents’ wishes. Guidance posters were displayed in each hospital between October 2025 and January 2026 to aid using the systems, and the doctors were surveyed once again (cycle 3) between 28/01/2026 and 04/02/2026 using the same questions.

**Results:**

Please note “easy” will be used where respondents rated the system easy or very easy. There were a total of 10 respondents in cycle 1: 70% found the now-previous system easy to access, only 50% found it easy to edit and 40% found they had missed tasks. Only 10% were opposed to trialling a new system. In cycle 2, there were a total of 22 respondents: 73% found the new system easy to access, 81% found it easy to edit and only 18% had missed tasks. 73% wished to keep the new system. In cycle 3, preliminary results show 10 respondents thus far: 100% found the new system easy to access and edit, only 10% had missed tasks and 100% wished to keep the new system. The system is now part of Standard Operating Procedure in North Glasgow.

**Conclusion:**

A new digital handover system replaced previous systems for each North Glasgow mental health hospital. The new system improved ease of access and editing as well as reducing the frequency of missed tasks for resident doctors. Displaying guidance posters further improved each of these categories and all doctors wished to keep the new system.

